# The Development of a Novel Headspace O_2_ Concentration Measurement Sensor for Vials

**DOI:** 10.3390/s23052438

**Published:** 2023-02-22

**Authors:** Xiao Chen, Hao Sun, Wei Huang, Jiayi Jin, Mingxu Su, Huinan Yang

**Affiliations:** School of Energy and Power Engineering, University of Shanghai for Science and Technology, Shanghai 200093, China

**Keywords:** measurement, O_2_, vial, concentration, sensor

## Abstract

In the process of manufacture and transportation, vials are prone to breakage and cracks. Oxygen (O_2_) in the air entering vials can lead to the deterioration of medicine and a reduction in pesticide effects, threatening the life of patients. Therefore, accurate measurement of the headspace O_2_ concentration for vials is crucial to ensure pharmaceutical quality. In this invited paper, a novel headspace oxygen concentration measurement (HOCM) sensor for vials was developed based on tunable diode laser absorption spectroscopy (TDLAS). First, a long–optical–path multi–pass cell was designed by optimizing the original system. Moreover, vials with different O_2_ concentrations (0%, 5%, 10%, 15%, 20%, and 25%) were measured with this optimized system in order to study the relationship between the leakage coefficient and O_2_ concentration; the root mean square error of the fitting was 0.13. Moreover, the measurement accuracy indicates that the novel HOCM sensor achieved an average percentage error of 1.9%. Sealed vials with different leakage holes (4, 6, 8, and 10 mm) were prepared to investigate the variation in the headspace O_2_ concentration with time. The results show that the novel HOCM sensor is non-invasive and has a fast response and high accuracy, with prospects in applications for online quality supervision and management of production lines.

## 1. Introduction

In recent years, the development of chemical and biosensors related to human and environmental health has become the hotspot of research [1]. For example, nanostructured material–based optical and electrochemical sensors were proposed to detect medicines in various biological and chemical samples [2,3,4,5]. In fact, the integrity of medicines’ packaging, e.g., vials, seriously affects the quality of medicines.

In order to ensure the stability and sterility of the medicines in vials, a vacuum or nitrogen (N_2_) gas environment is usually used in the pharmaceutical process to isolate the medicine from the environmental air [6,7]. However, vials are prone to breakage and cracks in the process of manufacture and transportation [8,9,10], leading to oxygen (O_2_) in the air entering the vials and reacting chemically with the medicine, which causes a great threat to pharmaceutical quality and safety. Therefore, the accuracy of headspace oxygen concentration measurement (HOCM) for vials is crucial to ensure pharmaceutical quality.

In recent years, researchers have developed a variety of methods for leakage detection. For example, Song et al. [11] employed a non–destructive high–voltage technique to detect pinhole leaks in packages; however, it was shown that the package contact surface area, thickness, and electrical conductivity significantly affected the detection. Patel et al. [12] performed a leak test of a lyophilized product in a parenteral vial package system by vacuum decay method, but the detection could have been prevented since leaks located below the product fill level might clog the flow of leaking gas. Moghimi et al. [13] applied injecting and vacuum dye penetration methods for the assessment of the package integrity of restorable flexible pouches with various sizes of micro–channels; however, the method depends on several physical parameters, including dye concentration, temperature, pressure differential, viscosity of the dye solution, and surface tension.

Tunable diode laser absorption spectroscopy (TDLAS) has attracted increasing attention for its high resolution, high sensitivity, and high stability and has been widely used in the field of gas detection [14,15,16,17,18,19]. Based on TDLAS, Cai et al. [20] developed a sensor for simultaneous measurements of the pressure and water vapor concentration in vials. This sensor does not require inert gas purges and calibration with a known gas. However, due to the water absorption of freeze–dried powder injections after low–temperature freeze drying, the leakage states of vials containing medicine cannot be accurately determined by this sensor in a short time after the leak. Luo et al. [21] established a signal reconstruction method based on discrete wavelet packet transform to effectively suppress stochastic noise and proposed a multiple linear regression–based oxygen concentration inversion scheme. However, the universality of this method for all signals needs to be further improved. Jenkins et al. [22] developed a sensor for detecting the headspace O_2_ concentration of vials. They experimented with demonstrating the accuracy of the sensor by employing a cylindrical glass test tube to simulate a vial. However, whether the sensor can be used to detect vials containing freeze–dried powder injections is yet to be confirmed. Zhu et al. [23] used the area of the spectral peak at half–height inversion of the headspace O_2_ concentration in vials. This method is tolerant to noise interference. Nevertheless, this work does not consider the nonlinear effects of vial rotation and real–time changes in the environment during the detection process. Lighthouse Instruments [24] provided a product for the HOCM of vials based on frequency modulation spectroscopy (FMS–760). However, due to the need for multiple rotational measurements to take the average value of the concentration and the complicated algorithm, the measurement time required for a single vial with this product is about 5 s, which is unable to meet the online rapid detection requirements of production lines.

Multi–pass optical cells provide the necessary long optical path to research weak trace–gas absorption features and are widely used in environmental monitoring [25], combustion processes [26], and medical diagnostics [27]. Liu et al. [28] summarized the design theory and research results of some mainstream types of multi–pass cells based on two mirrors and more than two mirrors in recent years and briefly introduced the application of some processed products. Das et al. [29] designed a compact multi–pass cell that focuses on achieving very long optical paths and demonstrated an optical path length of 50.31 m in a cell with 40 mm diameter mirrors spaced 88.9 mm apart. On this basis, they performed a second–harmonic wavelength modulation spectroscopy (WMS) measurement on weak carbon monoxide via TDLAS. Liu et al. [30] reported a highly sensitive hydrogen fluoride sensor based on the light–induced thermoelastic spectroscopy technique, and a Herriott multi–pass cell with an optical length of 10.1 m was selected to enhance the laser absorption. Krzempek et al. [31] developed a sensor for trace–gas detection of ethane (C_2_H_6_), which is based on a compact optical multi–pass cell and TDLAS, and the minimum detection limit is 740 pptv. It was found that the innovative ultra–compact, spherical multi–pass cell was capable of 459 passes between two separated mirrors with a 57.60 m effective optical path length. This work indicates that the combination of TDLAS and long–optical–path multi–pass cells is suitable for the detection of trace gases. However, there have been no studies on the application of this method for detection in vials.

In our previous work [32,33], we developed a leakage detection system for vials based on two–line TDLAS and proposed an O_2_ leakage coefficient *K* to judge the leakage states of the vials. However, the headspace O_2_ concentration of the vials cannot be accurately determined by the system. This paper further extends our previous work to develop a novel HOCM sensor for vials. Firstly, a long–optical–path multi–pass cell is designed based on the optical path of the original system, where the optical path length is increased significantly. In the meantime, a certain inclination angle of the base to suppress the optical noise caused by the etalon effect is designed. Secondly, vials with different O_2_ concentrations are measured by this optimized system to study the relationship between the leakage coefficient and O_2_ concentration. In addition, the accuracy of the optimized system measurement is verified by using standard O_2_ vials with known O_2_ concentrations. At the end of this paper, sealed vials with different leakage holes are studied in order to investigate the variation in the headspace O_2_ concentration in the vials with time.

## 2. Measurement System Development

### 2.1. The Novel HOCM Sensor

**Figure 1:**
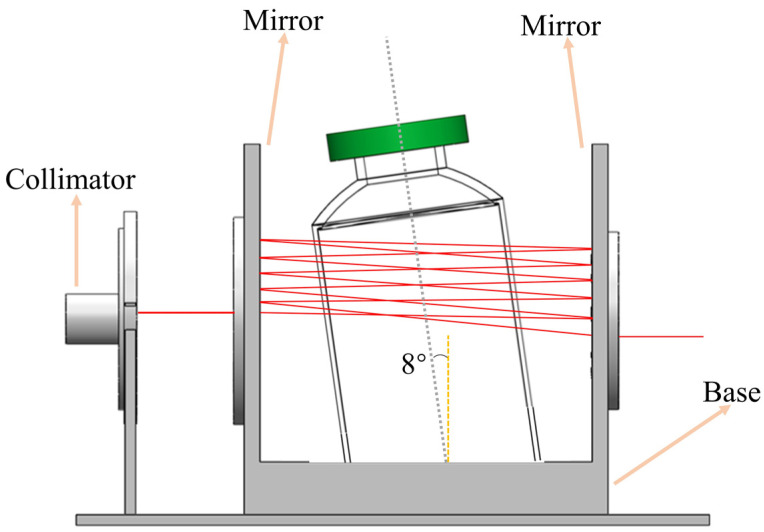
The long–optical–path multi–pass cell for vial detection.

In this paper, a long–optical–path multi–pass cell was designed, as shown in Figure 1. It consists of a collimating lens, a pair of silver–coated concave mirrors (with reflectivity of more than 96%), and a base. Through optical design, it can achieve about a 55 cm effective optical path capable of 11 passes between two mirrors separated by 5 cm.

Because the reflected light from different surfaces (such as the collimating lens and vial walls) can interfere with the measurement, it can lead to a complex pattern intensity of the Fabry–Pérot fringes, leading to optical noise [34,35,36]. Therefore, in this paper, the angle of the base was set to 8°, with the light incident at an angle of about 8° relative to the wall of the vial in order to suppress the optical noise of the etalon effects. It should be noted that the whole long–optical–path multi–pass cell is nitrogen purged in order to eliminate the influence of O_2_ in the ambient air.

**Figure 2:**
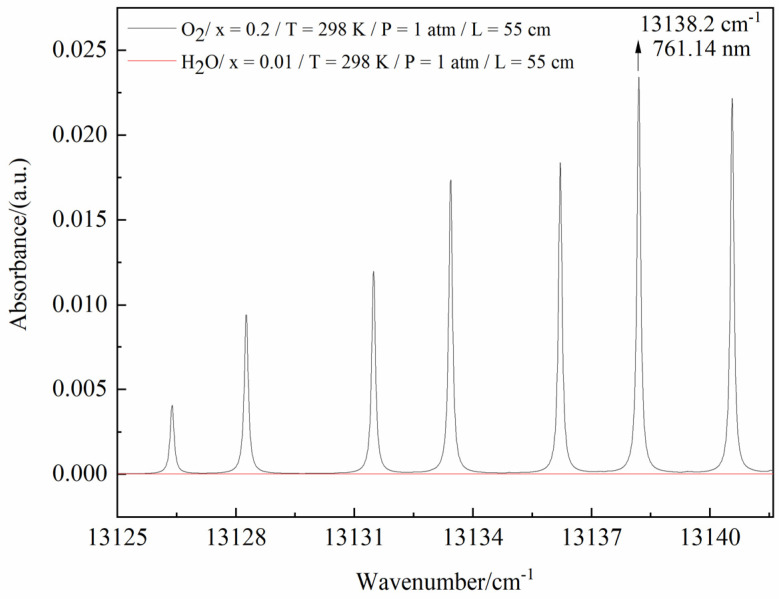
Simulated absorption spectra of O_2_ and H_2_O in the range of 13,125–13,142 cm^−1^.

O_2_ has many absorption lines around 760 nm [37]. According to the HITRAN 2020 database [38], the absorption spectra of O_2_ and water vapor near 760 nm (13,125–13,142 cm^−1^) were simulated under particular conditions (${\mathrm{Xo}}_{2}=0.2,\;X_{H_{2}O}=0.01$, T = 298 K, P = 1 atm, L = 55 cm), as shown in Figure 2. It is clearly shown that the absorption line at 13,138.2 cm^−1^ is the strongest line of O_2_ in the range, and there was almost no absorption for water vapor. Therefore, a diode laser (Eagleyard, EYP–DFB–0760) emission at the center wavelength of 761.14 nm was chosen as the laser source in this paper.

**Figure 3:**
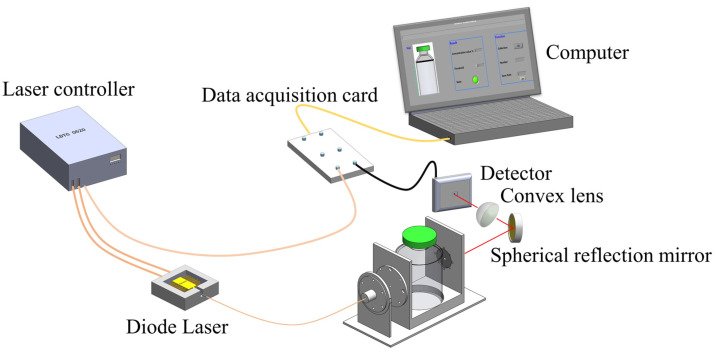
Diagram of the novel HOCM sensor for vials.

The diagram of the novel HOCM sensor for vials is shown in Figure 3. The temperature and current drive module (Wavelength Electronics, LDTC 0520) set the operating temperature and current of the diode laser to 18.7 °C and 100 mA, respectively, and then the center wavelength of the diode laser emission could be precisely set to 761.14 nm. The graphical programming software LabVIEW was used to output a saw–tooth signal with a repetition rate of 1000 Hz to scan through the selected O_2_ absorption lines repeatedly. After the laser passed through the multi–pass cell, the beams were reflected by a gold–coated spherical mirror (Edmund, 43–340) and further focused by a convex lens (Wuhan YouGuang, PCX–1801). The laser was finally detected by the photodetector (Thorlabs, PDA10CS) and converted into an electrical signal, which was transferred to the computer by the data acquisition card (NI, USB6361) for real–time acquisition and processing in LabVIEW. The measurement time required for a single vial is 0.2 s.

### 2.2. Calibration of O_2_ Concentration

In our previous work [32,33], we developed a leakage detection system for vials based on two–line TDLAS and proposed an O_2_ leakage coefficient to judge the leakage states of the vials. Nevertheless, the headspace O_2_ concentration of the vials cannot be accurately determined by the system. In the method of gas concentration detection based on TDLAS, the Voigt linear function is usually used for fitting in the direct absorption method, and the integral value of the spectral absorbance signal in the frequency domain is obtained from the linear fitting result; then, the gas concentration value is obtained. However, this method is not suitable for weak trace–gas detection. The wavelength modulation method can reduce the low–frequency noise of the system and improve the measurement sensitivity. However, it requires a lock–in amplifier to demodulate the harmonic signal, which makes the system complex, and the measurement is obtained as a relative value of the concentration change, which needs to be calibrated to obtain the gas concentration value. Therefore, this paper proposes calibrating the relationship between the leakage coefficient *K* and O_2_ concentration *C* through experiments so that the O_2_ concentration in the headspace of vials can be determined quickly and conveniently without complicated calculations.

The calibration system consists of two main parts: gas preparation and optical laser detection. The gas distribution is mainly composed of mass flow controllers (Seven Stars, CS–200), a gas tank, and a gas mixer. The two mass flow controllers are connected to the steel O_2_ and N_2_ tanks (the gas purity of the source tanks is 99.999% or more) through gas conduits. Additionally, the flow rates of O_2_ and N_2_ are controlled separately by control software to prepare different concentrations of O_2_ (0%, 5%, 10%, 15%, 20%, 25%) under ambient temperature and pressure conditions. While the gases are fully mixed in the gas mixer, the uniform gas mixture is then introduced into the vial through the gas conduit. After each experiment, the gas mixer is purged with pure N_2_ for two minutes to remove the residual gas and prevent it from affecting the measurement results. The optical laser detection part is the novel HOCM sensor for vials developed in this paper.

**Figure 4:**
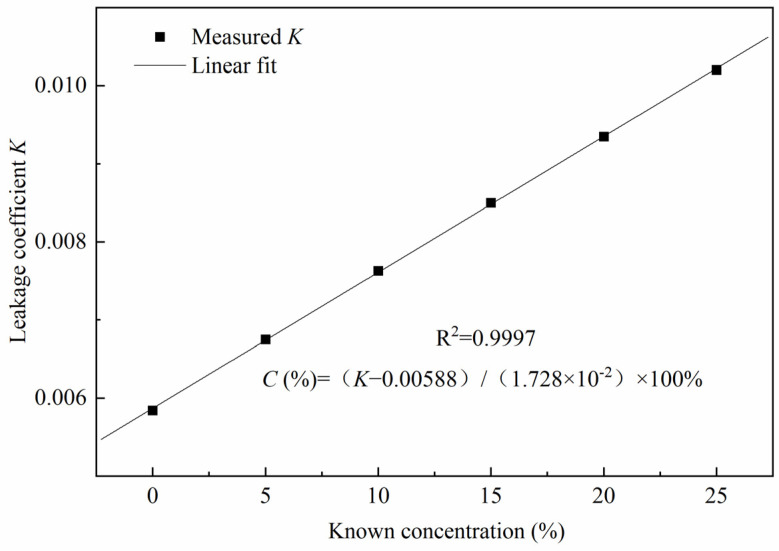
Leakage coefficient versus O_2_ concentration.

The calibration system was used to measure the *K* value of the vials under different concentrations of O_2_. In order to reduce the measurement error, vials with different O_2_ concentrations were each revolved 10 times, and the measurements were averaged. The average values of *K* obtained from the measurements were 0.00584, 0.00675, 0.00763, 0.0085, 0.00935, and 0.0102 for the vials with different O_2_ concentrations of 0%, 5%, 10%, 15%, 20%, and 25%, and the root mean square error of the fitting was 0.13. Figure 4 shows the result of the linear fit between the average value of *K* and the known concentrations as

*C* (%) = (*K* − 0.00588)/(1.728 × 10^−2^) × 100% (1)

The calculated R^2^ = 0.9997 indicates that the leakage coefficient *K* has an excellent linear relationship with the known O_2_ concentrations *C*.

### 2.3. Sensor System Measurement Accuracy Verification

In this paper, the measurement accuracy of the novel HOCM sensor was verified using four standard O_2_ vials (volume, 10 mL; bottom diameter, 22 mm; height, 50 mm) with known O_2_ concentrations. The four vials were evacuated and filled with a certified O_2_ gas mixture with concentrations of 0%, 7%, 14%, and 21% (the maximum allowable error of the O_2_ concentration was 1.6%), with N_2_ as a balance gas, and then melt–sealed to ensure the stability of the gas concentration in the vials. It should be noted that the laser light of the HOCM sensor only transmits through the upper part of the vial, while medicines are in the lower part of the vial. Therefore, medicines will not affect the measurement accuracy.

**Figure 5:**
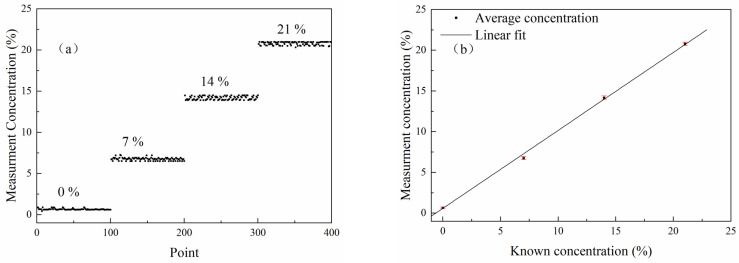
(**a**) O_2_ concentration values measured in four standard vials, the dots represent 100 measurement results for each known concentration; (**b**) measured average concentration values and the relationship of known concentration values.

The headspace O_2_ concentrations of the standard vials were measured separately using the novel HOCM sensor, and the results are shown in Figure 5a. The average values of 100 measurements for the four standard vials with different O_2_ concentrations of 0%, 7%, 14%, and 21% were 0.66%, 6.76%, 14.18%, and 20.8%, with standard deviations of 0.08%, 0.18%, 0.21%, and 0.20%, respectively. Note that the fluctuation at the concentration of 0% was significantly weaker than the others, which is due to the fact that, at the O_2_ concentration of 0%, the vial was filled with N_2_; this meant that the O_2_ concentration had less effect in the process of gas preparation, so the measurement results are better than those of the other vials.

Figure 5b presents the measured average concentration values and the relationships between the known concentration values. Notably, the average percentage error for the detected vials with different O_2_ concentrations using this novel HOCM sensor was 1.9%. There are many factors contributing to the average percentage error, including the interference effect caused by different standard vials and the optical noise interference, which changes when the laser enters the same vial from a different incident direction. Moreover, subtle differences in the thickness and smoothness of the vial’s surface can interfere with the measurement results to varying degrees [21].

## 3. Results and Discussions

**Figure 6:**
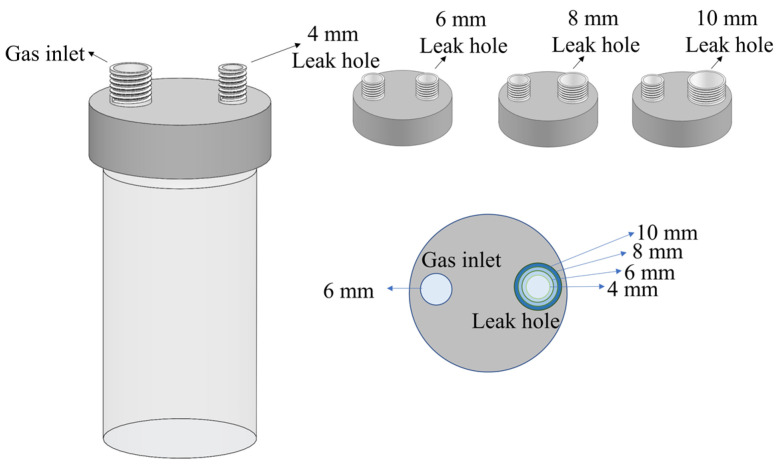
Schematic diagram of metal caps with different leakage holes.

The sensor was used to further investigate the variation in the O_2_ concentration in the headspace of the vials at different leakage degrees. For convenient processing, metal caps with leakage hole diameters of 4, 6, 8, and 10 mm were designed and processed. And they were sealed with sealant to prevent gas from escaping and causing measurement errors, as shown in Figure 6.

**Figure 7:**
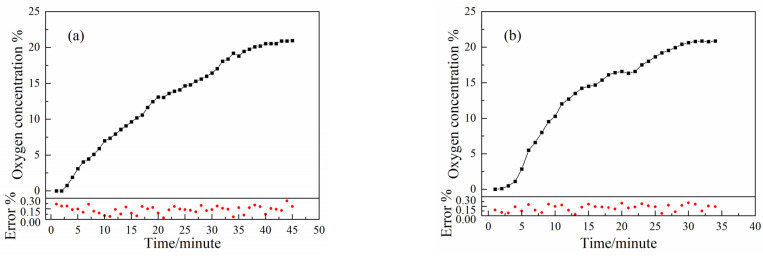

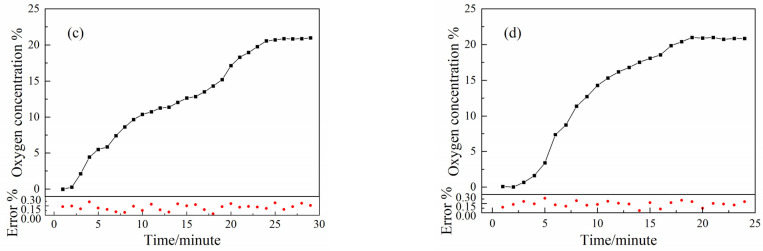
The variation in the O_2_ concentration with time after opening the leakage hole with different diameters, and the red dots indicate the measurement error: (**a**) 4 mm, (**b**) 6 mm, (**c**) 8 mm, and (**d**) 10 mm.

The sealed vial was placed on the base of the multi–pass cell with the gas inlet connected to the gas mixer through a gas conduit and the leakage hole sealed with a hemi–spherical nut. The mass flow controller passed N_2_ with 99.999% purity into the gas mixer and introduced N_2_ into the vial through the gas conduit. After the gas filled the vial, the inlet valve was closed and left to stand for one minute to prevent the flow of gas in the vial from causing measurement errors in the system. The headspace O_2_ concentration of the vial was measured by the novel HOCM sensor. The time zero point is the moment when the system starts the measurement. Additionally, the headspace O_2_ concentration of the vial was continuously measured to study the stability of the headspace O_2_ concentration before the leakage hole was opened. After two minutes, the leakage hole was opened to analyze the variation in the headspace O_2_ concentration in the vial with time until it was equal to the atmospheric O_2_ concentration. Then, the O_2_ concentration variation in the headspace of the vial with time was analyzed by using different leakage hole diameters. Figure 7 shows the variation in the O_2_ concentration in the vial with time for different leakage hole diameters every minute. The measurement error is also depicted.

The results show that the headspace O_2_ concentrations measured for different leakage hole diameters (4, 6, 8, and 10 mm) before the leakage hole opened were 0.015%, 0.13%, 0.26%, and 0.05%. These results were mainly due to the uneven quality of the surface of different vials and the influence of the environment, which caused the O_2_ concentration values measured before the leakage hole was opened to deviate to some extent. After the leakage hole was opened, due to the differences in the O_2_ concentration inside and outside the vials, the O_2_ in the environment quickly entered the vial through free diffusion and fully exchanged with the gas inside the vial. The time for the O_2_ concentration inside the vial to reach equilibrium with the environment decreased with the increase in the leakage hole diameter, with times of 43, 31, 26, and 21 min for leakage hole diameters of 4, 6, 8, and 10 mm, respectively. At this time, the headspace O_2_ concentration was 20.95%, 20.87%, 20.97%, and 20.86% due to the fluctuation in the O_2_ concentration in the environment, and the results for the measured concentration values also changed in a certain range, with the final concentration value tending to be the O_2_ concentration in the environment. This indicates that the larger the leakage hole diameter, the shorter the time required for the O_2_ concentration inside and outside the vial to reach equilibrium.

## 4. Conclusions

In this paper, a novel HOCM sensor for vials was developed, combining TDLAS and a long–optical–path multi–pass cell. Firstly, a long–optical–path multi–pass cell was designed based on the optical path of the original system, where a significant increase in the optical path length was achieved. Additionally, we selected a certain inclination angle of the base to suppress the optical noise caused by the etalon effect. Secondly, we proposed calibrating the relationship between *K* and the known O_2_ concentration *C* through experiments so that the O_2_ concentration in the headspace of the vial can be determined quickly and conveniently without complex calculations. Moreover, the measurement accuracy based on vials with known O_2_ concentrations (0, 7, 14, and 21%) indicates that this novel sensor achieved an average percentage error of 1.9%. Thirdly, sealed vials with different leakage degrees were measured in real time, and the time taken for the O_2_ concentrations both inside and outside the vials to reach equilibrium was investigated. It is also shown that the novel HOCM sensor developed in this paper has the advantages of a fast response and high accuracy and is also non–invasive, demonstrating prospects in applications for online quality supervision and for the management of production lines. However, the vials with other properties, such as shape, material, or color, will be investigated in our future work so that the developed sensor can be better applied to different actual situations. As an optical measurement method for sealed containers, this method can also be applied to other fields, such as foodstuffs and packaging.

## 5. Patents

This work has reported a patent, “A leak detection device for vials combining TDLAS and gas multi–pass cell (Patent number 202210270467.2).”

## Data Availability

The data presented in this study are available on request from the corresponding author.

## References

[1] Imran M., Ahmed S., Abdullah A.Z., Hakami J., Chaudhary A.A., Rudayni H.A., Khan S.U., Khan A., Basher N.S. (2022). Nanostructured material-based optical and electrochemical detection of amoxicillin antibiotic. Luminescence.

[2] Krishnan S.K., Singh E., Singh P., Meyyappan M., Nalwa H.S. (2019). A review on graphene-based nanocomposites for electrochemical and fluorescent biosensors. RSC Adv..

[3] Shi L., Wang Y., Ding S., Chu Z., Yin Y., Jiang D., Luo J., Jin W. (2017). A facile and green strategy for preparing newly-designed 3D graphene/gold film and its application in highly efficient electrochemical mercury assay. Biosens. Bioelectron..

[4] Majdinasab M., Mitsubayashi K., Marty J.L. (2019). Optical and Electrochemical Sensors and Biosensors for the Detection of Quinolones. Trends Biotechnol..

[5] Shen L., Wang H., Chen P., Yu C., Liang Y., Zhang C. (2016). The analytical determination and electrochemiluminescence behavior of amoxicillin. J. Food Drug Anal..

[6] Ditter D., Mahler H.C., Roehl H., Wahl M., Huwyler J., Nieto A., Allmendinger A. (2018). Characterization of surface properties of glass vials used as primary packaging material for parenterals. Eur. J. Pharm. Biopharm..

[7] Schaut R.A., Peanasky J.S., DeMartino S.E., Schiefelbein S.L. (2014). A new glass option for parenteral packaging. PDA J. Pharm. Sci. Technol..

[8] Chow E.J., Kitaguchi B., Trier M. (2012). Effects of subzero temperature exposure and supercooling on glass vial breakage: Risk management and other applications in cold chain distribution. PDA J. Pharm. Sci. Technol..

[9] Iacocca R.G., Toltl N., Allgeier M., Dong X., Foubert M., Hofer J., Peoples S., Shelbourn T. (2010). Factors affecting the chemical durability of glass used in the pharmaceutical industry. AAPS Pharmscitech.

[10] Milton N., Gopalrathnam G., Craig G.D., Mishra D.S., Roy M.L., Yu L. (2007). Vial breakage during freeze-drying: Crystallization of sodium chloride in sodium chloride-sucrose frozen aqueous solutions. J. Pharm. Sci..

[11] Song Y.S., Gera M., Jain B., Koontz J.L. (2014). Evaluation of a non-destructive high-voltage technique for the detection of pinhole leaks in flexible and semi-rigid packages for foods. Packag. Technol. Sci..

[12] Patel J., Mulhall B., Wolf H., Klohr S., Guazzo D.M. (2011). Vacuum Decay Container Closure Integrity Leak Test Method Development and Validation for a Lyophilized Product-Package System. PDA J. Pharm. Sci. Technol..

[13] Moghimi N., Park S. (2017). Leakage assessment of flexible pouches using dye penetration test with correlation to modeled bacterial aerosol challenge test. Food Sci. Biotechnol..

[14] Lundin P., Cocola L., Lewander M., Olsson A., Svanberg S. (2012). Non-intrusive headspace gas measurements by laser spectroscopy—Performance validation by a reference sensor. J. Food Eng..

[15] Luo L., Li T., Deng J., Zhao R., Wang J. (2022). An improved WMS-2f/1f spectral fitting method using orthogonal test in initial parameters selection. Sensors.

[16] Wang F.P., Chang J., Wang Q., Wei W., Qin Z.G. (2017). TDLAS gas sensing system utilizing fiber reflector based round-trip structure: Double absorption path-length, residual amplitude modulation removal. Sens. Actuators A.

[17] Liu X., Ma Y. (2022). Tunable diode laser absorption spectroscopy based temperature measurement with a single diode laser near 1.4 μm. Sensors.

[18] Neethu S., Verma R., Kamble S.S., Radhakrishnan J.K., Krishnapur P.P., Padaki V.C. (2014). Validation of wavelength modulation spectroscopy techniques for oxygen concentration measurement. Sens. Actuators B.

[19] Lu H., Zheng C., Zhang L., Liu Z., Song F., Li X., Zhang Y., Wang Y. (2021). A remote sensor system based on TDLAS technique for ammonia leakage monitoring. Sensors.

[20] Cai T.D., Wang G.S., Cao Z.S., Zhang W.J., Gao X.M. (2014). Sensor for headspace pressure and H_2_O concentration measurements in closed vials by tunable diode laser absorption spectroscopy. Opt. Lasers Eng..

[21] Luo Q., Song C., Yang C., Gui W., Sun Y., Jeffrey Z. (2020). Headspace oxygen concentration measurement for pharmaceutical glass bottles in open-path optical environment using TDLAS/WMS. IEEE Trans. Instrum. Meas..

[22] Jenkins T.P., Berg T. Diode Laser Absorption Sensor for Detecting Oxygen in Head Space of Vials. Proceedings of the 2008 IEEE Sensors.

[23] Zhu G.F., Yang C.H., Zhu H.Q., Gui W.H. (2017). Oxygen concentration detection and calibration method improvement in pharmaceutical vial based on wavelength modulation spectroscopy. Spectrosc. Spect. Anal..

[24] Laser-Based Headspace Analysis|Lighthouse Instruments. http://lighthouseinstruments.com.

[25] Silver J.A., Wood W.R. Miniature gas sensor for monitoring biological space environments. Proceedings of the 2002 International Symposium on Optical Science and Technology.

[26] Goldenstein C.S., Spearrin R.M., Jeffries J.B., Hanson R.K. (2017). Infrared laser-absorption sensing for combustion gases. Prog. Energy Combust. Sci..

[27] Gianella M., Sigrist M.W. (2010). Infrared spectroscopy on smoke produced by cauterization of animal tissue. Sensors.

[28] Liu Y., Ma Y. (2023). Advances in multipass cell for absorption spectroscopy-based trace gas sensing technology. Chin. Opt. Lett..

[29] Das D., Wilson A.C. (2011). Very long optical path-length from a compact multi-pass cell. Appl. Phys. B.

[30] Liu X., Qiao S., Han G., Liang J., Ma Y. (2022). Highly sensitive HF detection based on absorption enhanced light-induced thermoelastic spectroscopy with a quartz tuning fork of receive and shallow neural network fitting. Photoacoustics.

[31] Krzempek K., Jahjah M., Lewicki R., Stefanski P., So S., Thomazy D., Tittel F.K. (2013). CW DFB RT diode laser based sensor for trace-gas detection of ethane using a novel compact multipass gas absorption cell. Appl. Phys. B.

[32] Yang H., Chen J., Luo X., Liu C., Qi D., Xin X., Su M. (2019). Leakage detection of closed vials based on two-line water-vapor TDLAS. Measurement.

[33] Zhang Y., Wu W., Yang H., Li C., Tao J., Kan R. (2020). Optimization of leakage detection system for vials based on two-line tunable diode laser absorption spectroscopy. Spectrochim. Acta Part A Mol. Biomol. Spectrosc..

[34] Persson L., Andersson F., Andersson M., Svanberg S. (2007). Approach to optical interference fringes reduction in diode laser absorption spectroscopy. Appl. Phys. B.

[35] He J., Song C., Luo Q., Lan L., Yang C., Gui W. (2020). Noise-robust self-adaptive support vector machine for residual oxygen concentration measurement. IEEE Trans. Instrum. Meas..

[36] Zhu G., Hu X., Zhu H., Hu E., Zhu J. (2018). The multi-beam interference suppression for measuring penicillin vial’s oxygen concentration based on tunable diode laser absorption spectroscopy. Spectrosc. Spect. Anal..

[37] Shen S., He J., Wang X. (2022). Relationship between harmonic line shape and temperature and pressure for wavelength modulation spectroscopy. Opt. Eng..

[38] Gordon I.E., Rothman L.S., Hargreaves R.J., Hashemi R., Karlovets E.V., Skinner F.M., Conway E.K., Hill C., Kochanov R.V., Tan Y. (2022). The HITRAN2020 molecular spectroscopic database. J. Quant. Spectrosc. Radiat. Transf..

